# Effect of an individualised nutritional intervention on gestational diabetes mellitus prevention in a high-risk population screened by a prediction model: study protocol for a multicentre randomised controlled trial

**DOI:** 10.1186/s12884-021-04039-2

**Published:** 2021-08-24

**Authors:** Chenjie Zhang, Lulu Wang, Wenguang Sun, Lei Chen, Chen Zhang, Hong Li, Jiale Yu, Jianxia Fan, Huijuan Ruan, Tao Zheng, Dongling Wu, Shaojing Li, Huan Lu, Man Wang, Ben W. Mol, Hefeng Huang, Yanting Wu

**Affiliations:** 1 International Peace Maternity and Child Health Hospital, School of Medicine, Shanghai Jiao Tong University, 910, Hengshan Rd, Shanghai, 200030 China; 2 Shanghai Key Laboratory of Embryo Original Diseases, Shanghai, 200030 China; 3 Obstetrics and Gynecology Hospital, Institute of Reproduction and Development, Fudan University, Shanghai, 200011 China; 4 Department of Gynecology, Xinhua Hospital, Shanghai Jiao Tong University, Shanghai, 200092 China; 5 Department of Obstetrics and Gynecology, Shanghai Jiao Tong University-Affiliated Sixth People’s Hospital of Fengxian Branch, Shanghai, 201499 China; 6 Department of Obstetrics and Gynaecology, Monash University, Clayton, Victoria Australia

**Keywords:** Study protocol, Gestational diabetes mellitus, Nutritional interventions, Randomised controlled trial, Prediction model, Three-day food records

## Abstract

**Background:**

The ability of a preventive nutritional intervention to reduce the morbidity of gestational diabetes mellitus (GDM) remains controversial. We aim to assess whether GDM can be prevented by an individualised nutritional intervention in pregnant women who are at high risk for the disease based on a prediction model.

**Methods/design:**

A multicentre randomised controlled trial was designed to assess the efficacy of an individualised nutritional intervention for the prevention of GDM in a high-risk population screened by a novel prediction model in the first trimester. Pregnant women evaluated to be at high risk for GDM by the prediction model at less than 14 gestational weeks will be included. Women with pre-existing chronic diseases, including pregestational diabetes, or who are currently prescribed medicines that affect glucose values will be excluded. Allocation to intervention/control at a ratio of 1:1 will be conducted by a computerized randomisation system. The intervention group will complete 3-day food records and receive 3 individualised nutritional consultations with professional dieticians before the oral glucose tolerance test. The primary intention of the intervention is to promote a long-term healthy dietary pattern and prevent excessive gestational weight gain throughout pregnancy. The control group will complete 3-day food records at designated gestational weeks and receive standard antenatal care according to local health care provisions. The primary outcome is the incidence of GDM according to the criteria of the International Association of Diabetes and Pregnancy Study Group (IADPSG). A sample of 464 participants will provide 80% power to detect a 30% reduction in GDM incidence (α = 0.05 two tailed, 10% dropout). A total of 500 participants will be recruited.

**Discussion:**

To date, this is the first randomised controlled trial aimed to evaluate the protective effect of an individualised nutritional intervention against GDM based on a logistic regression prediction model. Eligibility is not limited to obese women or singleton pregnancies, as in previous studies. This pragmatic trial is expected to provide valuable information on early screening and effective GDM prevention methods.

**Trial registration number:**

ChiCTR, ChiCTR1900026963. Registered 27 October 2019.

**Supplementary Information:**

The online version contains supplementary material available at 10.1186/s12884-021-04039-2.

## Background

Gestational diabetes mellitus (GDM) is a common, pregnancy-specific disorder diagnosed at 24–28 gestational weeks. The East Asian population has high GDM susceptibility, with an incidence rate of 11.7% (range: 4.5–25.1%) [1]. The current diagnostic criteria for GDM are inconsistent worldwide. The international medical community, including that in China, adopts the results of the 75 g oral glucose tolerance test (OGTT) at 24–28 gestational weeks as diagnostic criteria as recommended by the IADPSG and the American Diabetes Association (ADA) (fasting venous glucose ≥5.1 mmol/L, 1-h value ≥10.0 mmol/L, 2-h value ≥8.5 mmol/L) [2, 3]. GDM can exert several adverse effects in both mothers and children, including polyhydramnios, macrosomia and neonatal hypoglycaemia [4, 5]. Moreover, reasonable evidence has indicated that exposure to an intrauterine hyperglycaemic environment leads to an increased risk of chronic health issues in filial generations, such as cardiovascular diseases, diabetes mellitus, and obesity [6–8]. Despite the adverse effects of GDM, referral to professional nutritionists for individualised lifestyle modifications before the OGTT is infrequently implemented in current clinical practice in China.

Previous studies have indicated that lifestyle modification strategies addressing healthy eating and/or physical activity can effectively prevent type 2 diabetes mellitus in nonpregnant populations [9]. Nutritional patterns in early pregnancy have been suggested to be associated with GDM development [10]. Counselling and behavioural interventions have been reported to be effective in limiting excess gestational weight gain (GWG), which is associated with a decreased risk of GDM [11–13]. As a first-line strategy for GDM management, nutritional interventions mainly involve a dietician’s personalised diet prescription in accordance with nutritional intake guidelines during pregnancy [14]. A prospective study with a small sample size (*n* = 50) suggested that GWG in obese women (body mass index (BMI) ≥ 30 kg/m^2^) can be reduced through the implementation of 10-h dietary consultations (gain of 6.6 kg in the intervention group vs gain of 13.3 kg in the control group, *p* = 0.002), thus reducing the incidence of GDM (0% in the intervention group vs 10% in the control group) [15]. The conclusions of an RCT conducted in nine European countries with 150 participants with BMI ≥ 29 kg/m^2^ support the application of early healthy eating interventions in obese pregnant women, as evidenced by lower GWG and lower fasting glucose in the intervention group [16]. Another prospective randomised controlled trial with a larger sample size (*n* = 269) conducted in Finland showed that obese pregnant women (BMI ≥ 30 kg/m^2^) who received individualised counselling on physical activity, diet, and weight gain control from trained nurses and dieticians had a lower prevalence of GDM (13.9% in the intervention group vs 21.6% in the control group, *p* = 0.044) [17]. Nevertheless, the UPBEAT study from the UK indicated that behavioural intervention in obese pregnant women (BMI ≥ 30 kg/m^2^) was not adequate to prevent GDM (25.0% in the intervention group vs 26.0% in the standard care group, *p* = 0.68) [18]. Currently, the level of evidence on whether nutritional intervention can prevent GDM is considered to be low to very low since it is challenging to implement these interventions and monitor measurement error and diet complexity under free-living circumstances [19, 20]. On the other hand, previous studies mostly targeted obese pregnant women with singleton pregnancies or those with a previous history of GDM [14, 15, 17]. As stated in a study conducted in America, nearly one-third of GDM patients (29.3%) were underweight or normal weight [21]. Currently, it remains unproven whether nutritional intervention can prevent GDM in a more general population, such as normal-weight women, women with multiple pregnancies or women with fasting blood glucose equal to or higher than 5.1 mmol/L in the early conception period. Therefore, modifiable risk factors and pragmatic interventions need to be identified.

Given the promising potential to relieve the social and economic burden of GDM, comprehensive and high-quality clinical research with adequate power to identify evidence-based preventive strategies is urgently warranted. Therefore, we present a multicentre, open-label and parallel-group randomised study protocol to explore whether an individualised nutritional intervention can effectively reduce the prevalence of GDM in pregnant women who have been identified by a prediction model in the first trimester to be at high risk for the disease. Previous studies have built various risk evaluation models for GDM taking different clinical risk factors into consideration; however, these models lack external validation or applicability for clinical practice [22–24]. Prior to the design of this study, we developed a novel logistic regression prediction model via advanced machine learning, the establishment details of which have been published previously [25]. The model selected 7 out of 73 clinical indicators: age, blood triglyceride level, fasting blood glucose and HbA1c levels in early pregnancy, family history of diabetes in first-degree relatives, multiple pregnancy and previous history of GDM. These indicators were considered to be sensitive predictors of GDM as early as the initiation of pregnancy in previous studies [22–24, 26–30]. With a solid theoretical foundation, this pragmatic trial will provide reliable clinical evidence of the efficacy of an individualised nutritional intervention to prevent GDM among high-risk populations.

### Objective and hypothesis

The aim of this trial is to evaluate the effectiveness of an individualised nutritional intervention for the prevention of GDM among high-risk pregnant women screened by a novel risk prediction model in the first trimester. We hypothesise that providing individualised nutritional consultations at least once a month before the OGTT for high-risk pregnant women will reduce the prevalence of GDM and improve maternal and offspring perinatal outcomes.

## Methods and analysis

### Study design and setting

**Fig. 1:**
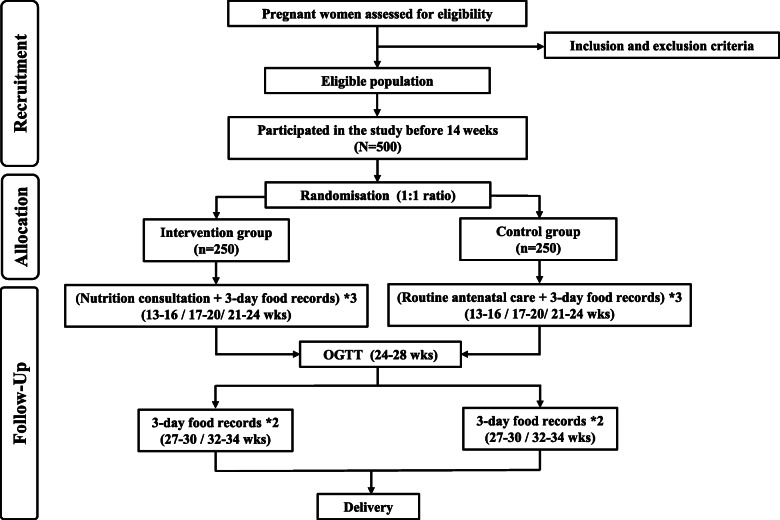
Flowchart of participant recruitment, randomisation and follow-up

The proposed study is a multicentre, open-label, parallel-group randomised trial. A total of 500 eligible women will be recruited from 3 different tertiary hospitals, including +/− 300 from the International Peace Maternity and Child Health Hospital, +/− 100 from Xinhua Hospital, and +/− 100 from Shanghai Fengxian District Central Hospital. A flowchart of participant recruitment, randomisation and follow-up is shown in Fig. 1 (see Additional file 1).

### Participants

Women in early pregnancy who attend the antenatal clinics of three research centres will be identified by the GDM risk prediction model mentioned above. According to the multiple logistic regression model we previously established, the predictive probability of GDM development in our study population will be calculated with the formula 1/[1 + exp.(−β)], in which β equals (− 14.2334 + (0.0681*age) + (0.5005* blood triglyceride level in the first trimester, mmol/L) + (2.8165* fasting blood glucose level in the first trimester, mmol/L) + (1.1062*family history of diabetes in first-degree relatives) + (1.6925* HbA1c in the first trimester, %) + (0.4349*multiple pregnancy) + (2.6181* previous history of GDM). The area under the receiver operating characteristic curve of this model was 0.77 (95% CI 0.76–0.78). The optimum threshold probability was 0.13 with 59% sensitivity and 82% specificity when the Youden index was set at 0.41 [24]. Consequently, pregnant women with a calculated risk probability of GDM equal to or higher than 0.13 in the first trimester are considered high risk. Among them, pregnant women at less than 14 gestational weeks who are able to understand and provide informed consent and are ready to receive antenatal care and deliver at one of the research centres will be eligible to participate in our study.

We will exclude individuals if they have been diagnosed with the following disorders: pre-pregnancy essential hypertension, renal disease, HIV, hepatitis B or C, cardiac disease, thalassemia, cystic fibrosis, systemic lupus erythematosus or any other autoimmune disease, thyroid disease requiring medication, pregestational diabetes (including patients diagnosed with diabetes before conception; fasting blood glucose ≥7.0 mmol/L or HbA1c ≥ 6.5% in the first trimester; typical hyperglycaemic symptoms or hyperglycaemic crisis with optional blood glucose ≥11.1 mmol/L), or current prescription of medicines, such as metformin and glucocorticoids, that affect blood glucose values.

### Recruitment and randomisation

The research staff will verbally inform eligible pregnant women that the hospital is currently conducting a multicentre clinical trial to identify better preventive strategies for GDM and that they are eligible to participate. If they fully understand and are willing to join the study, they will meet the research staff in person at the hospital, sign an informed consent form and officially participate in the project prior to 14 gestational weeks. Baseline data, including sociodemographic characteristics and medical and maternal history, will be collected by standardised questionnaires. Each participant will be given a sequential study number by research staff. Subsequently, their basic information will be entered into an internet-based and password-protected clinical research management system (ResMan Research Manager, supported by West China Hospital, Sichuan University, China), which will automatically generate a research code and randomly allocate participants to the intervention group or the control group at a ratio of 1:1. The research assistants responsible for data collection, biostatisticians and data analysts will be masked to study group allocation. Given the nature of the intervention, the participants and investigators will be aware of the assignment.

### Dietary intervention

For the intervention group, one-to-one nutritional counselling with the same dietician will be arranged 3 times before the OGTT (13–16 w, 17–20 w, 21–24 w). The average contact time with a dietician will be 30 min for each subject per visit. The primary intention of the nutritional intervention is to promote a long-term healthy dietary pattern and prevent excessive GWG throughout pregnancy, thereby potentially lowering the prevalence of GDM and resulting in better perinatal outcomes. Qualified dieticians from three centres will receive specific training with the aim of establishing a uniform set of intervention standards for the study. The proposed dietary strategies are based primarily on the International Federation of Gynaecology and Obstetrics (FIGO) endorsed guidelines for pregnant women and the Diagnosis and Therapy Guideline for Pregnancy with Diabetes Mellitus (2014) from the Chinese Medical Association (CMA) and Chinese Dietary Guidelines (2016) from the Chinese Nutrition Society [31–33]. Macronutrient intake composition will be recommended to be in compliance with current clinical practice for patients diagnosed with GDM. Protein, fat and carbohydrates should account for 15–20%, 25–30%, and 45–50%, respectively, of the total energy intake. Low-glycaemic index (GI) foods (target GI ≤ 50) are recommended as carbohydrate sources due to their associations with appropriate GWG and ameliorated maternal glucose tolerance [34]. More specifically, the counselling will focus mainly on GWG management, food preparation techniques, meal timing, and proportions of whole grains, dark green leafy vegetables, nuts, fruits and lean protein consumption. Refined carbohydrate foods and other sugar-rich products will be restricted.

Compared with common dietary consultations, the greatest advantage of the intervention in this study lies in self-administered 3-day food records, a valid resource for nutritionists to pinpoint problems precisely and optimise dietary patterns individually. At enrolment, participants will be instructed by a trained research practitioner on how to record their diet diary in detail. A consecutive 3-day food record should cover 2 weekdays and 1 weekend day excluding special occasions (e.g., banquets, business trips, or tours). To obtain information that can represent participants’ usual dietary intakes, they are asked to truthfully record all foods and beverages consumed as accurately as possible. The food diary consists of consumption time, food category, preparation techniques and the accurate amount of raw ingredients in mixed dishes. To ensure a more precise and unified quantification of foods, participants will receive an 18-page colouring booklet that graphically shows commonly consumed local foods with serving sizes and different measurement tools. It is suggested that participants take pictures of foods for which it is difficult to estimate portion sizes themselves, which provides intuitive evidence for objective postcompletion revision by nutritionists. Before each appointment with a dietician, women assigned to the intervention group will be asked to complete a 3-day food record in advance. To ascertain whether their dietary patterns changed after the OGTT, 2 additional diet diaries will be required to be completed at 27–30 and 32–34 gestational weeks. During the study visits, food records will be entered by dieticians into nutritional analysis software (NutriStar Software, Zhending, China) based on the China Food Composition Tables (2018) [35], which will help to calculate energy and nutrient intake for in-depth quantified evaluation. Then, a tailored dietary prescription will be developed as mentioned above for further management in the intervention group. Subsequent visits will be scheduled by dieticians or research staff at the end of each consultation. Strategies have been established for retention of participants enrolled. A specific trained research staff will be responsible for follow-up and direct communications with all the participants at each trial centre. The research staff will not only remind them in person to fill in 3-day food records or to visit a dietician at designated gestational weeks, but also emphasize the benefits of the study to encourage them to complete follow-up. For convenience, study visits will be arranged in parallel with standard antenatal clinic visits. Additionally, priority access to ultrasound examinations during pregnancy will be provided. Adherence to the intervention is anticipated to be assessed comprehensively taking into consideration nutritional clinic attendance as required and the number of qualified 3-day food records. If needed, comparisons between nutrition prescriptions and actual diet records will be performed.

### Standard care

All the participants in both groups will attend standard antenatal care at their trial centre and will be provided with regular treatments if diagnosed with GDM. The control group will fill in five 3-day food records at designated gestational week periods (13–16 w, 17–20 w, 21–24 w, 27–30 w, 32–34 w) but will not be scheduled to see a study-specific dietician as part of the study. However, the control group participants will be allowed to visit nutritionists themselves for usual antenatal healthy diet advice in accordance with local health care provisions.

### Withdrawal of participants

The participants will be informed clearly by investigators at enrolment that they may withdraw from the study at any period for any reason without penalty. Written consent will be obtained if the participant agrees to undergo continued follow-up for associated clinical outcomes after withdrawal.

### Participant timeline

The time schedule of recruitment, intervention, and outcome assessments is presented in Table 1 (see Additional file 2). All pregnant women will take a blood test assessing fasting glucose, HbA1c, and blood triglyceride levels in the first trimester. The number of foetuses will be determined by ultrasound in early pregnancy. After the required data are obtained, the recruiting staff will screen every single pregnant woman at antenatal clinics using the prediction model to determine their eligibility for the study and initiate recruitment as specified previously. Written informed consent, baseline information, and baseline blood pressure plus anthropometric measurements (body weight, height) will be obtained at recruitment. Fasting body weight measured in light clothing without shoes as well as blood pressure will be followed up at 1-month intervals during antenatal visits. The first 3-day food record will be distributed at first contact. Women in the intervention group will receive 3 nutritional consultations that will be paired with 3-day food records at 13–16, 17–20, and 21–24 gestational weeks before the OGTT and submit 2 more diaries after (27–30 w, 32–34 w). The participants in the control group will return their diaries directly to research staff 5 times throughout gestation (13–16 w, 17–20 w, 21–24 w, 27–30 w, 32–34 w). All the participants in both arms will be required to take a 75 g OGTT at 24–28 gestational weeks, the results of which will be the primary end point.

**Table 1:**
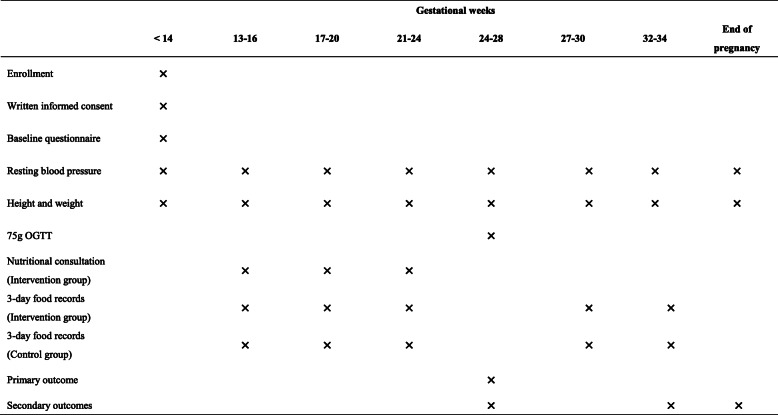
Data collection schedule

### Proposed outcome measurement

#### Primary outcome

GDM diagnosis via a 75 g OGTT at 24–28 gestational weeks will be the primary end point, which is defined as meeting or exceeding any one of the following plasma glucose values in the one-step screening: fasting plasma glucose ≥5.1 mmol/L; 1-h value ≥10.0 mmol/L; and 2-h value ≥8.5 mmol/L (adapted from the IADPSG and the ADA [2, 3]).

#### Secondary outcomes [36]

*Maternal-* GWG, hypertensive disorders of pregnancy (gestational hypertension, preeclampsia), placental abruption, preterm/prelabor/ rupture of membranes (P/PROM), mode of birth (vaginal delivery/operational vaginal delivery/elective or emergency caesarean section), blood loss during birth, postpartum haemorrhage, adherence to the intervention, requirement for insulin therapy, total daily insulin dose, and maternal mortality will be assessed. GWG will be recorded to the nearest 0.1 kg as the difference between self-reported pre-pregnancy weight and the last measurement of weight prior to delivery in the hospital [37]. Gestational hypertension will be defined as de novo hypertension (≥ 140/90 mmHg) on two occasions at least 4 h apart after 20 gestational weeks without proteinuria or other biochemical, systematic, or severe features in a woman who was normotensive preconception. Preeclampsia will be diagnosed when gestational hypertension is accompanied by any of the following presentations: new-onset proteinuria (≥ 0.3 g/24 h), thrombocytopenia (PLT ≤ 100,000 × 10^9/L), renal insufficiency, impaired liver function or pulmonary oedema [38]. Maternal biochemical outcomes include the OGTT results (fasting, 1-h and 2-h glucose values), fasting plasma insulin level, and insulin resistance calculated by homeostatic model assessment 2 (HOMA2-IR) at 24–28 gestational weeks and average fasting glucose level, HbA1c level, haemoglobin concentration, and lipid profile [low-density lipoprotein (LDL), high-density lipoprotein (HDL), triglycerides, total cholesterol] at the 3rd trimester.

*Neonatal-* Infant sex; gestational age at birth (based on estimated date of delivery); Apgar score; birth length; birth weight assessed for macrosomia (≥ 4000 g), low birth weight (< 2500 g), large for gestational age (LGA), and small for gestational age (SGA); preterm birth (PTB, < 37 weeks); shoulder dystocia; bone fracture; bronchial plexus injury; neonatal hypoglycaemia; neonatal respiratory distress; jaundice for which treatment is warranted; neonatal intensive care unit (NICU) admission; congenital anomalies; livebirth; miscarriage (< 20 weeks); still birth (≥ 20 weeks); and neonatal death (within the first 28 days after delivery) will be assessed. LGA and SGA will be defined as a birth weight above the 90th or below the 10th percentiles of newborns of the same gestational age and sex, respectively, in accordance with the INTERGROWTH-21st growth standards published in 2014 [39]. Neonatal hypoglycaemia refers to venous glucose levels lower than 2.6 mmol/L within the first 48 h after birth.

*Dietary-* Daily average energy intake, macronutrient intake (proteins, fats, carbohydrates), vitamin intake (vitamin A, vitamin D, vitamin E, vitamin C, vitamin B1, vitamin B2, niacin, and folic acid), mineral intake (calcium, phosphorus, iron, and zinc), and dietary fibre intake (g) will be assessed.

#### Data collection

At the time of enrolment, data on demographic characteristics, socioeconomic status, smoking habit, alcohol consumption, self-reported morbidity, menstrual and obstetric history, first-degree family history of diabetes, hypertension, stroke, hyperlipidaemia and obesity will be collected using a baseline questionnaire. Gestational weeks, height, weight and resting blood pressure will be measured and recorded by research staff at the time of enrolment and at each follow-up visit during prenatal care, as shown in Table 1. BMI is equal to the weight in kilograms divided by height in metres squared (kg/m^2^). Maternal age; pre-pregnancy weight and BMI; parity; prior GDM; family history of diabetes; number of foetuses; prenatal diagnoses; laboratory results of fasting glucose level, HbA1c level, and triglycerides at the 1st trimester; OGTT results of fasting, 1-h and 2-h glucose values and fasting plasma insulin at 24–28 weeks gestation; and average fasting glucose level, HbA1c level, haemoglobin concentration, and lipid profile at the 3rd trimester during 32–34 gestational weeks will be collected from the outpatient electronic medical record (EMR). Data on maternal and neonatal secondary outcomes stated above will be obtained from inpatient EMRs and will be confirmed by research clinicians. In the intervention arm, poor adherence to the intervention will be considered as failure to complete 3 nutritional consultations or at least 4 sets of qualified 3-day food records at the appropriate times. If food quantification information is missing from a 3-day food record, it will be recognised as invalid. The criteria for diet diaries will be equally applicable to the control arm. Dietary statistics will be processed and calculated automatically by NutriStar Software based on 3-day food records provided by the participants.

### Statistical methods

#### Sample size calculation

The incidence of GDM among high-risk women determined by the prediction model is approximately 30% in accordance with the IADPSG criteria on the basis of our unpublished data. Assuming a clinically important 30% reduction in GDM incidence in the intervention arm and allowing for 10% dropout, the calculated sample size of 464 subjects (232 per group) would provide 80% power with a two-tailed alpha error of 0.05. Thus, we aimed to recruit 500 women in total (250 per group), anticipating that at least 450 participants would complete the trial.

### Statistical analysis

Intention-to-treat principles will be followed in statistical analyses. Categorical variables will be expressed as counts with percentages. Normally distributed data will be presented as the means with standard deviations (SDs), while nonnormally distributed data will be reported as medians with interquartile ranges (IQRs). Comparisons of significant differences between groups will be performed by Student’s *t*-test for continuous variables with normal distributions, the nonparametric Kruskal–Wallis test for nonnormally distributed data, and the Pearson chi-square test for categorical data. With regard to repeated measurements throughout gestation (e.g., weight), the results will be presented separately for each timepoint. For binary endpoints, risk ratios and risk differences with 95% confidence intervals will be calculated by binomial regression. All analyses will be performed unadjusted and adjusted for appropriate baseline values.

#### Data management and confidentiality

All paper versions of the materials will be stored in a designated locked cabinet, while electronic data will be saved in the password-secured ResMan Research Manager system. Double data entry will be performed by a full-time trained clerk and a supervisor. Original information from pen-and-paper baseline questionnaires and 3-day food records will be collected and processed under the instructions of clinicians and nutritionists of the study. Data entered incorrectly will be examined and corrected by the supervisor after confirmation with the participants or their obstetric records. Any revision of the original data will be tracked in detail.

Participants’ personal information or any data will remain anonymous and be kept securely throughout the study. Unauthorized access to the database or disclosure is not allowed at any trial centre. All staff members are responsible for strict confidentiality of information at all times.

#### Data monitoring and auditing

The Trial Steering Committee (TSC) is composed of CZ, SJL, DLW, BWM, HFH and YTW, who will be responsible for study oversight and overall conduct. A Data Monitoring Committee (DMC) consisting of four members will be set up for on-site audits and data quality control. The DMC will be independent from the sponsor, and there will be no competing interests. Two audits for each centre will be performed: at the midpoint and at the end of the trial. Emphasis will be placed on whether recruitment, data collection, nutritional intervention and follow-ups are conducted and updated according to the protocol. Moreover, the quality of self-administered 3-day food records will be comprehensively assessed for both completeness and precision. Validity of original data source will be verified by randomly selected samples. A formal report will be created, which will provide useful instructions not only for problem solving in due time but also for statistical analyses in the future. No interim analysis has been planned for the primary or secondary outcomes. In terms of adverse events (AEs), diet interventions are safe considering that evidence on related harms was very limited in general [11], but any unintended occurrence of AEs associated with the study interventions will be reported to the TSC and recorded in detail by the staff member.

### Ethics approval and consent to participate

Ethical approval was obtained from the Medical Research Ethics Committees of the International Peace Maternity and Child Health Hospital (25 October 2019, GKLW 2019–11), Shanghai Fengxian District Central Hospital (19 November 2019, 2019-KY-11) and Xinhua Hospital (1 June 2020, XHEC-C-2020-086). All study procedures will comply with the Declaration of Helsinki. All participants will sign a written informed consent before participating in the trial. There were no protocol amendments.

### Dissemination

Meetings will be held periodically for progress promotion and quality control. The completion of the study and publication of the results will be attributed to all study collaborators. The participants will receive feedback through open-access publications about the ultimate results of this trial. To reach the widest possible dissemination of the findings, open access publications in high impact journals, oral and poster presentations with interests in GDM prevention will be performed nationwide and internationally.

## Discussion

The pathogenesis of gestational diabetes remains unclear due to the heterogeneity of the at-risk population and multiple risk factors, including individual lifestyles, special physiological environment preconception or during pregnancy, genetic susceptibility, etc. [40, 41]. No consensus has been reached universally on early screening of GDM in at-risk populations or standardised effective strategies to prevent the disease. Instead of setting simple criteria (e.g., prior GDM, obesity) for eligibility as was done in previous research, high-risk pregnant women eligible for this clinical trial will be detected by a novel 7-feature logistic regression prediction model developed by our study group. Although similar prediction models for GDM have been reported before, few of them have been put into clinical practice for further validation. It is anticipated that this model will be able to recognise women at risk of GDM to a greater extent as soon as they become pregnant.

Nonetheless, several limitations of our study design should be noted. First, participants and practitioners will not be blinded to group allocation, which might introduce bias. Another limitation that cannot be ruled out is that the control arm will still have access to general information on healthy eating, which might affect the outcomes. Despite these limitations, our study will determine whether a regular individualised nutritional intervention that is easy to implement in the 1st and 2nd trimesters before the OGTT is capable of reducing the incidence of GDM as well as composite adverse effects among high-risk populations. If successful, the findings of the study can be transferred to clinical practice rapidly, which will provide valuable information to improve guidelines on early screening and effective prevention methods of GDM.

## Supplementary Information

**Additional file 1.**

**Additional file 2.**

**Additional file 3.**

## Data Availability

Not applicable.

## Abbreviations

GDM: Gestational diabetes mellitus
IADPSG: International Association of Diabetes and Pregnancy Study Group
OGTT: Oral glucose tolerance test
ADA: American Diabetes Association
GWG: Gestational weight gain
BMI: Body mass index
HbA1c: Haemoglobin A1c
HIV: Human immunodeficiency virus
FIGO: International Federation of Gynaecology and Obstetrics
CMA: Chinese Medical Association
GI: Glycaemic index
PROM: Prelabor rupture of membranes
PPROM: Preterm prelabor rupture of membranes
PLT: Platelet
HOMA2-IR: Homeostatic model assessment 2 - Insulin resistance
LDL: Low-density lipoprotein
HDL: High-density lipoprotein
LGA: Large for gestational age
SGA: Small for gestational age
PTB: Preterm birth
NICU: Neonatal intensive care unit
EMR: Electronic medical record
TSC: Trial Steering Committee
DMC: Data Monitoring Committee
AEs: Adverse events
SDs: Standard deviations
IQRs: Interquartile ranges
