# Supplementary material for: Effect of an individualised nutritional intervention on gestational diabetes mellitus prevention in a high-risk population screened by a prediction model: study protocol for a multicentre randomised controlled trial
Source: BMC Pregnancy Childbirth. 2021 Aug 24;21:586. doi: 10.1186/s12884-021-04039-2 (PMC8385988; doi:10.1186/s12884-021-04039-2)
Supplement: Supplementary file 1 — Additional file 1. [file 12884_2021_4039_MOESM1_ESM.pptx]

## Slide 1
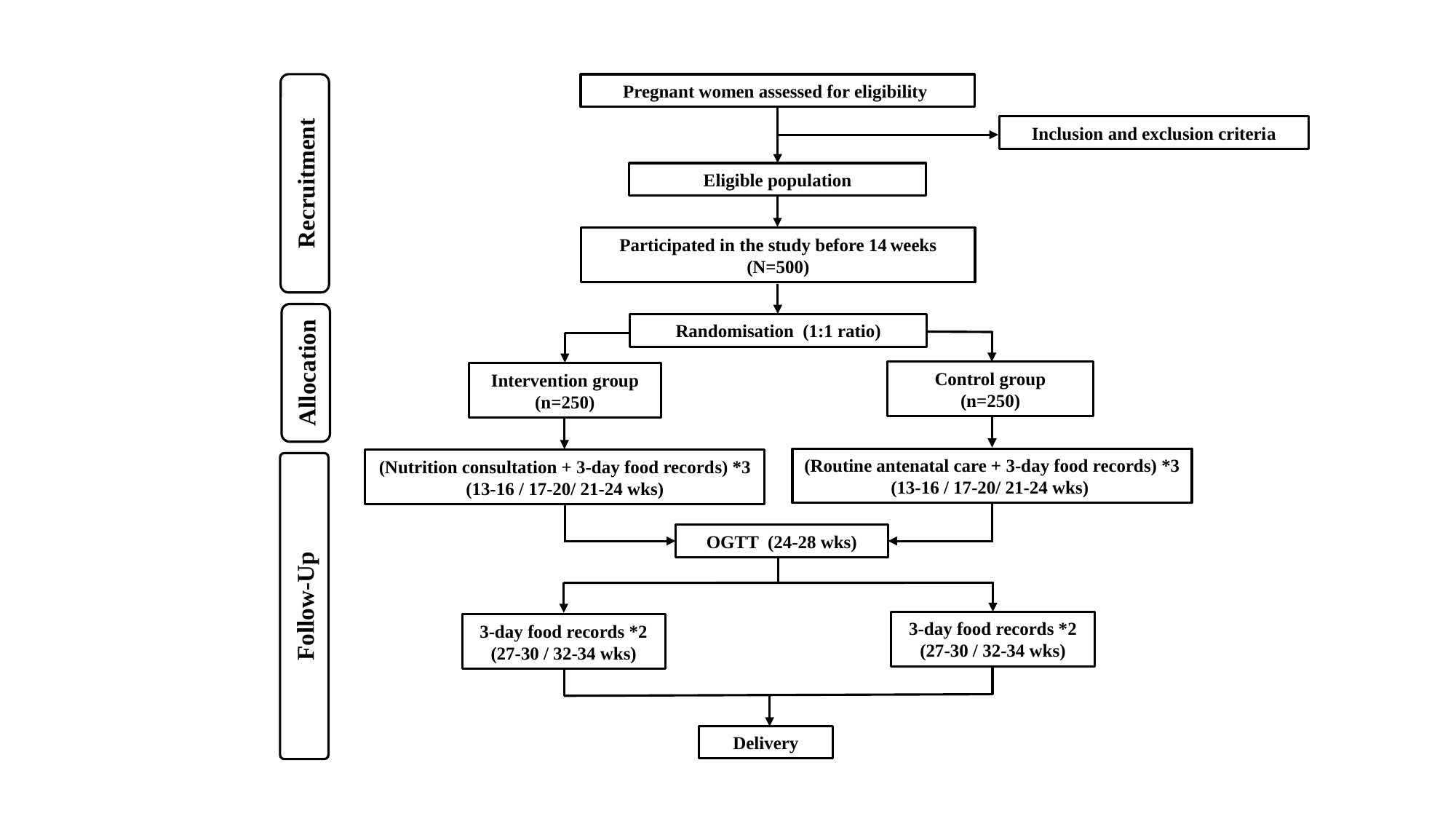

Recruitment
Pregnant women assessed for eligibility
Inclusion and exclusion criteria
Eligible population
Participated in the study before 14 weeks
(N=500)
Allocation
Randomisation (1:1 ratio)
Control group
(n=250)
Intervention group
(n=250)
(Routine antenatal care + 3-day food records) *3
(13-16 / 17-20/ 21-24 wks)
(Nutrition consultation + 3-day food records) *3
(13-16 / 17-20/ 21-24 wks)
Follow-Up
OGTT (24-28 wks)
3-day food records *2
(27-30 / 32-34 wks)
3-day food records *2
(27-30 / 32-34 wks)
Delivery
