# Supplementary material for: Effect of an individualised nutritional intervention on gestational diabetes mellitus prevention in a high-risk population screened by a prediction model: study protocol for a multicentre randomised controlled trial
Source: BMC Pregnancy Childbirth. 2021 Aug 24;21:586. doi: 10.1186/s12884-021-04039-2 (PMC8385988; doi:10.1186/s12884-021-04039-2)
Supplement: Supplementary file 2 — Additional file 2. [file 12884_2021_4039_MOESM2_ESM.docx]

**Table 1. Data collection schedule**

|  | | | | | **Gestational weeks** | | | | |
| --- | --- | --- | --- | --- | --- | --- | --- | --- | --- |
|  | **< 14** | **13-16** | **17-20** | **21-24** | | **24-28** | **27-30** | **32-34** | **End of pregnancy** |
| Enrollment | **×** |  |  |  | |  |  |  |  |
| Written informed consent | **×** |  |  |  | |  |  |  |  |
| Baseline questionnaire | **×** |  |  |  | |  |  |  |  |
| Resting blood pressure | **×** | **×** | **×** | **×** | | **×** | **×** | **×** | **×** |
| Height and weight | **×** | **×** | **×** | **×** | | **×** | **×** | **×** | **×** |
| 75g OGTT |  |  |  |  | | **×** |  |  |  |
| Nutritional consultation (Intervention group) |  | **×** | **×** | **×** | |  |  |  |  |
| 3-day food records  (Intervention group) |  | **×** | **×** | **×** | |  | **×** | **×** |  |
| 3-day food records  (Control group) |  | **×** | **×** | **×** | |  | **×** | **×** |  |
| Primary outcome |  |  |  |  | | **×** |  |  |  |
| Secondary outcomes |  |  |  |  | | **×** |  | **×** | **×** |
